# On the temperature dependence of H-*U*_iso_ in the riding hydrogen model

**DOI:** 10.1107/S2053273314010626

**Published:** 2014-05-28

**Authors:** Jens Lübben, Christian Volkmann, Simon Grabowsky, Alison Edwards, Wolfgang Morgenroth, Francesca P. A. Fabbiani, George M. Sheldrick, Birger Dittrich

**Affiliations:** a Institut für Anorganische Chemie, Georg-August-Universität, Tammannstrasse 4, D-37077 Göttingen, Germany; b School of Chemistry and Biochemistry, Stirling Highway 35, WA-6009 Crawley, Australia; c Bragg Institute, Australian Nuclear Science and Technology Organisation, Locked Bag 2001, Kirrawee DC, NSW 2232, Australia; d Institut für Geowissenschaften, Abteilung Kristallographie, Goethe-Universität, Altenhöferallee 1, 60438 Frankfurt am Main, Germany; e GZG, Abteilung Kristallographie, Georg-August Universität, Goldschmidtstrasse 1, 37077 Göttingen, Germany; f Institut für Anorganische und Angewandte Chemie, Martin-Luther-King-Platz 6, 20146 Hamburg, Germany

**Keywords:** riding hydrogen model, QM/MM computations, neutron diffraction, invariom refinement, Hirshfeld-atom refinement, synchrotron radiation

## Abstract

The temperature dependence of H-*U*_iso_ in *N*-acetyl-l-4-hydroxyproline monohydrate is investigated. Imposing a constant temperature-independent multiplier of 1.2 or 1.5 for the riding hydrogen model is found to be inaccurate, and severely underestimates H-*U*_iso_ below 100 K. Neutron diffraction data at temperatures of 9, 150, 200 and 250 K provide benchmark results for this study. X-ray diffraction data to high resolution, collected at temperatures of 9, 30, 50, 75, 100, 150, 200 and 250 K (synchrotron and home source), reproduce neutron results only when evaluated by aspherical-atom refinement models, since these take into account bonding and lone-pair electron density; both invariom and Hirshfeld-atom refinement models enable a more precise determination of the magnitude of H-atom displacements than independent-atom model refinements. Experimental efforts are complemented by computing displacement parameters following the TLS+ONIOM approach. A satisfactory agreement between all approaches is found.

## Abstract 2

The temperature dependence of hydrogen *U*_iso_ and parent *U*_eq_ in the riding hydrogen model is investigated by neutron diffraction, aspherical-atom refinements and QM/MM and MO/MO cluster calculations. Fixed values of 1.2 or 1.5 appear to be underestimated, especially at temperatures below 100 K.

## 1. Introduction   

The riding hydrogen model is widely used in refining small-molecule X-ray diffraction data. Three positional and one isotropic displacement parameter can be constrained to a ‘parent atom’ that the H atom is ‘riding’ on, improving the data-to-parameter ratio and ensuring a chemically meaningful geometry. Alternatively, a single isotropic displacement parameter per riding H atom can be included in the least-squares refinement model while still constraining hydrogen positional parameters.

Predicted H-atom positions usually lead to comparable figures of merit to a free refinement of H-atom positional parameters. This holds even for high-quality X-ray data, extending far into reciprocal space, since the scattering contribution of hydrogen is small and limited in resolution. Therefore predicted positions, *e.g.* by *SHELXL*  (Sheldrick, 2008 ▶), have also been used for ‘invariom’ (Dittrich *et al.*, 2004 ▶) aspherical-atom refinements (Schürmann *et al.*, 2012 ▶; Pröpper *et al.*, 2013 ▶). Such H-atom treatment, in combination with elongating *X*—H vectors to bond distances computed by quantum chemical optimizations of model compounds, provides structures of high quality from conventional diffraction data.

As stated above, the riding hydrogen model can include constraints for isotropic hydrogen displacement parameters. Ratios of 1.2 and 1.5 of H-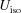 with respect to the 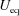 of the parent atom are being used in most refinement programs today. These ratios had been empirically derived for use with room-temperature data. However, most of today’s data sets are collected at temperatures of 100 K or lower, making full use of reduced thermal motion, *e.g.* to reduce the bias of anisotropic displacement parameters on bond distances (Busing & Levy, 1964 ▶). We will show that the ratio of H-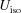/*X*-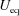 is temperature dependent, which indirectly follows from Bürgi & Capelli (2000 ▶). Therefore constant H-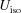 multipliers are inaccurate; the simple remedy of using temperature-dependent multipliers is proposed herein.

Taking into account the temperature dependence of riding hydrogen treatments of H-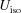 is a detail of increasing importance in X-ray diffraction, as experimental data quality is improving with modern detectors and X-ray sources. Taking the effect into account allows removal of a resolution-dependent systematic error that would otherwise only affect low-resolution data, which is where the hydrogen scattering contributes. While the effect of underestimating H-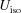 might seem unimportant when only looking at the *R* factor (which is practically unchanged), the effect can be frequently detected when aspherical scattering factors,^1^ which take into account bonding and lone-pair electron-density distribution, are used for least-squares refinement of positional and atomic displacement parameters (ADPs).^2^ For charge-density studies, where the aim is to adjust the scattering factor *via* multipole parameters to the X-ray data, the anisotropic description of atomic displacements should be used. Hydrogen ADPs are usually estimated in such studies. Munshi *et al.* (2008 ▶) have compared competing approaches for such estimates, and the *SHADE* (simple hydrogen anisotropic displacement estimator) server (Madsen, 2006 ▶) is the approach most frequently used for that purpose today. Since the focus of this work is the most frequently used isotropic treatment of hydrogen displacements, we will not discuss the anisotropic description here.

## 2. Experimental   

Single crystals of the compound *N*-acetyl-l-4-hydroxyproline monohydrate (NAC

**Figure 1:**
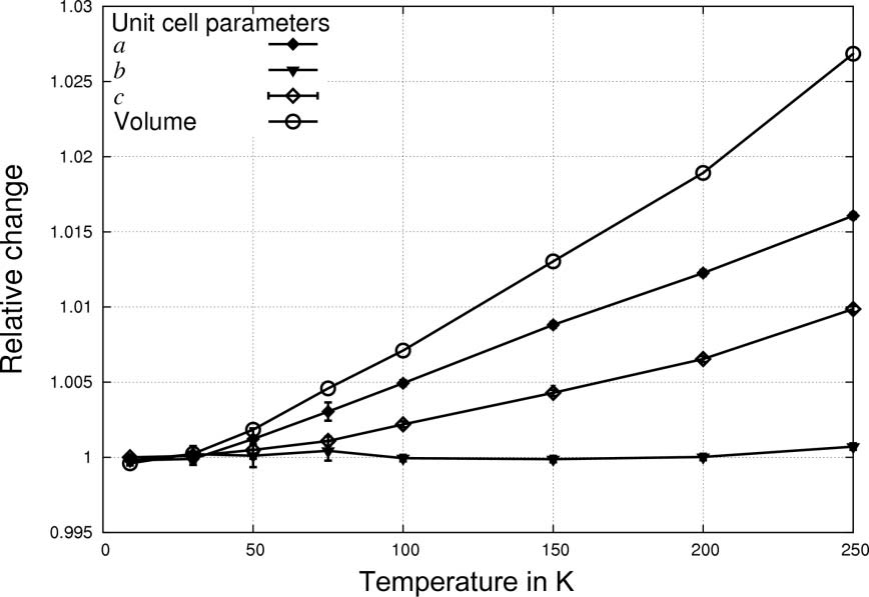
Temperature dependence of the lattice constants of the X-ray data of *N*-acetyl-l-hydroxyproline monohydrate. Unit-cell parameters and volume are normalized to the lowest data point at 9 K. E.s.d’s are also plotted (but may not be visible when small). Connecting lines are only guidelines for the eye.

H_2_O) were grown by slow evaporation of saturated solutions prepared in hot acetone. Crystals grow to sizes suitable for neutron diffraction. A series of multi-temperature X-ray diffraction data collections^3^ at 9, 30, 50 and 75 K on the same specimen with dimensions of 0.34 × 0.28 × 0.28 mm (0.5 mm pinhole) were collected at the DORIS beamline D3 at the HASYLAB/DESY synchrotron in Hamburg. The experimental setup consisted of an Oxford Diffraction open-flow helium gas-stream cooling device, a Huber type 512 four-circle diffractometer and a 165 mm MAR CCD detector. A wavelength of 0.5166 Å and a detector distance of 40.3 mm were chosen, allowing a high resolution of 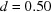
 Å or 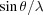 of 1.0 Å^−1^ to be reached with a single detector setting. The *XDS* program (Kabsch, 2010 ▶) was used for data integration and scaling. Standard deviations of the unit-cell parameters (Fig. 1 ▶) were obtained by calculating the variance of intermediate cells during integration.

A detector correction (Johnas *et al.*, 2006 ▶) was applied to properly correct for the effect of oblique incidence (Wu *et al.*, 2002 ▶) on the measured intensities. An empirical absorption correction was not performed at this short wavelength; Friedel opposites were merged. The structural model, cell settings but not the atom notation of the original structure determination by Hospital *et al.* (1979 ▶) as given in the CIF file of the Cambridge Structural Database refcode NAHYPL were used as input. Preliminary least-squares refinements were initialized with this model and performed with the program *SHELXL* (Sheldrick, 2008 ▶).

Data sets at 100, 150 and 200 and 250 K were collected on an Xcalibur S diffractometer equipped with an Mo 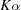 sealed tube. Here an analytical absorption correction was performed following the method of Clark & Reid (1995 ▶) as implemented in the program *CRYSALIS RED* (Oxford Diffraction Ltd, 2006 ▶) employed for data reduction; Friedel mates were not merged. A second specimen was used for these four higher temperatures. High-resolution data (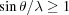) were again measured with the exception of the data set at 250 K.

**Figure 2:**
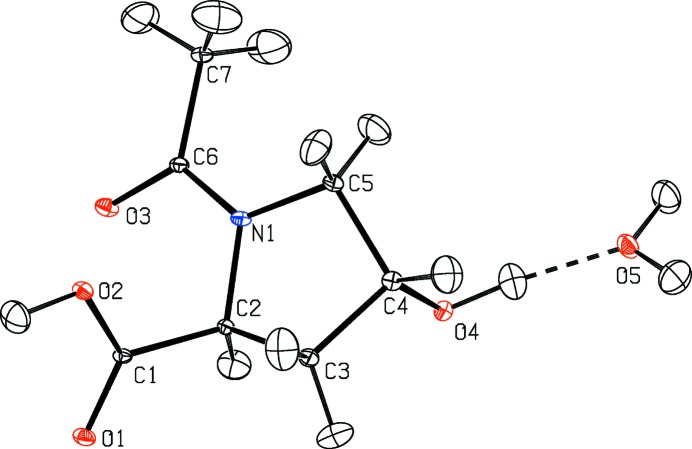
ADPs of *N*-acetyl-l-hydroxyproline monohydrate from neutron diffraction at *T* = 9 K. Ellipsoids at 50% probability (Burnett & Johnson, 1996 ▶).

Neutron diffraction data were collected at the OPAL reactor on the Koala beamline at ANSTO, the Australian Nuclear Science and Technology Organization, in Lucas Heights, Australia. Laue neutron data were collected for a single specimen 1.8 × 1.4 × 0.5 mm using an unmonochromated thermal neutron beam on KOALA (Edwards, 2011 ▶) at  9, 150, 200 and 250 K. Each data set comprises 16, 12, 12 and ten images (each exposure = 42 min) accumulated on the image plate and from which intensities were extracted using *LaueG* (Piltz, 2011 ▶) employing unit-cell dimensions from the corresponding X-ray determination. The *CRYSTALS* program (Betteridge *et al.*, 2003 ▶) was used for the refinement of positions and ADPs for all atoms. An isotropic extinction parameter was required at 9 K due to good crystal quality and comparably large specimen size for the neutron data. CCDC 977814–977817 contain the supplementary crystallographic information for the neutron data. These files can be obtained free of charge from the Cambridge Crystallographic Data Centre *via* http://www.ccdc.cam.ac.uk/data_request/cif. A depiction of the molecule with its atomic numbering scheme and anisotropic ADPs at 9 K from neutron diffraction is shown in Fig. 2 ▶.

## 3. Methods   

H-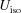/*X*-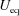 ratios reported here were derived from four independent methods. Benchmark results for NAC

H_2_O were obtained from multi-temperature neutron diffraction. Values for the hydrogen ADPs from multi-temperature single-crystal X-ray diffraction evaluated with the independent-atom model (IAM) cannot reach the accuracy achievable by neutron diffraction. To improve the physical significance of ADPs and their accuracy from X-ray diffraction (Jelsch *et al.*, 1998 ▶; Dittrich *et al.*, 2008 ▶), we therefore performed aspherical-atom refinements [either Hirshfeld-atom (Jayatilaka & Dittrich, 2008 ▶) or invariom refinement (Dittrich *et al.*, 2004 ▶), see below]. QM/MM and MO/MO quantum mechanical cluster calculations (for details of how to run such computations see Dittrich *et al.*, 2012 ▶) yield normal modes within the ‘molecular Einstein approximation’. These were combined with a TLS fit in the TLS+ONIOM approach (Whitten & Spackman, 2006 ▶) and were subsequently converted to give anisotropic ADPs for H atoms. Such computations were performed to complement the experimental results (see §3.3).

### 3.1. Aspherical-atom refinements   

Two types of aspherical-atom refinements were performed: in invariom refinement (Dittrich *et al.*, 2005 ▶, 2006 ▶, 2013 ▶) the molecular electron-density distribution is reconstructed from Hansen/Coppens’ multipole-model parameter values (Hansen & Coppens, 1978 ▶) as tabulated in the generalized invariom database (Dittrich *et al.*, 2013 ▶). In Hirshfeld-atom refinement (HAR) (Jayatilaka & Dittrich, 2008 ▶) the electron density of the asymmetric unit is obtained by a single-point energy calculation.

*Invariom refinements*. For structure models based on invariom refinements a least-squares refinement of positions and displacement parameters was carried out using the program *XDLSM* of the *XD2006* package (Volkov *et al.*, 2006 ▶). The program *INVARIOMTOOL* (Hübschle *et al.*, 2007 ▶) was used to set up *XD* system files for that purpose. Refinement was against 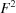 with a *SHELXL*-type weighting scheme, and the 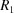 factor was calculated for all reflections with 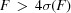. Crystallographic details are given in Table 1 ▶.

CCDC 990102–990109 contain the supplementary crystallographic data for the X-ray structures. CIF files including intensities are only provided for the invariom refinements, since the same intensities were also used for HAR.

Scattering factors, their local atomic site symmetry and invariom names as well as the model compounds these were derived from are given in Table 2 ▶. H-atom positions were initially calculated with *SHELXL*. In invariom refinement the *X*—H bond distances were then elongated during initial scale-factor refinement to optimized bond distances of the respective model compound for the invariom assigned to the H atom. This new H-atom position was then constrained to have the same shift as the parent *X* atom. Only 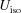 values were freely refined. This procedure was followed because it is also feasible when conventional data of lower resolution than the data studied here are available. Moreover, idealized H-atom positions provided better input for the MO/MO cluster computations (see §3.3), since idealized positions facilitate reaching convergence. Ratios of hydrogen 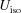 to 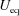 of the parent atom were then averaged for H atoms sharing the same invariom name using the program *APD-TOOLKIT*.^4^ For direct comparison with HAR, free refinement of H atoms was also performed and the results obtained (not shown) are very similar.

*Hirshfeld-atom refinement*. In Hirshfeld refinement the electron density from single-point energy calculations is used and partitioned into atomic contributions using Hirshfeld’s fuzzy boundary partitioning scheme (Hirshfeld, 1977 ▶). Fourier transform (Jayatilaka, 1994 ▶) then gives aspherical atomic scattering factors. Atomic positions and ADPs are adjusted to best fit the experimental data using these scattering factors. In an improved implementation of HAR in the quantum crystallography program *TONTO* (Jayatilaka & Grimwood, 2003 ▶), cycles of molecular electron-density calculations, aspherical-atom partitioning and least-squares refinement are now iterated to convergence in an automatic manner (Capelli *et al.*, 2014 ▶). The Hartree–Fock method was used in combination with the basis set cc-pVTZ (Dunning, 1989 ▶). A supermolecule cluster approach was chosen to calculate a wavefunction for both molecules of the asymmetric unit for use in HAR (Woinska *et al.*, 2014 ▶). The structural model used in HAR included individual positional parameters and isotropic ADPs for H atoms. Ratios of hydrogen 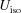 and 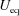 of the parent atom were again averaged for H atoms sharing the same invariom name. As expected and shown before for three urea derivatives (Checińska *et al.*, 2013 ▶), both types of aspherical-atom models, the Hansen & Coppens multipole model and HAR, give similar figures of merit and anisotropic ADPs of the non-H atoms with experimental X-ray data.

### 3.2. Neutron diffraction   

As mentioned before, the H-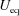/*X*-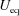 ratios from neutron diffraction provide benchmark values for this study. One of the advantages of neutron diffraction is that the scattering lengths of the elements that correspond to atomic scattering factors in X-ray diffraction are constant. Stewart (1976 ▶) demonstrated that 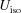 and 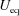 from single-crystal X-ray and neutron diffraction differ, and that 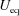 will be in between the arithmetic and geometric mean of the diagonal elements of the mean-square displacement matrix. Since we are interested in the *ratio* of hydrogen 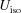 and 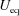 of the parent atom, conventional least-squares adjustment can nevertheless provide relative reliable experimental estimates of atomic motion at a particular temperature. Equivalent isotropic displacements H-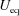 5 (orthorhombic system) were obtained both by geometric and by arithmetric averaging the diagonal elements of the matrix of the anisotropic displacements of H atoms (Fischer & Tillmanns, 1988 ▶), and both give the same ratios within the estimated uncertainty. In contrast to the deposited structural model, refinements were evaluated without using split-atom sites to model rotational disorder in the methyl group above 150 K. Structural models are given in the supporting information.^6^

### 3.3. Theoretical computations   

A quantum mechanical cluster computation was performed to complement the experimental results. The computation was initiated using the experimental geometry from invariom refinement at the lowest temperature of 9 K with idealized hydrogen positions and elongated *X*—H distances. The method/basis set for optimizing these model compounds was B3LYP/D95++(3df,3pd). The utility program *BAERLAUCH* (Dittrich *et al.*, 2012 ▶) was used to generate a cluster of 17 asymmetric units packed around a central unit. The water solvent molecule was optimized together with the main molecule. Preliminary QM/MM calculations [HF/6-31G(d,p):UFF] ensured that this cluster size leads to convergence and is suitable to reproduce experimental ADPs at low temperature. Calculations to obtain final results employed the MO/MO basis-set combination B3LYP/cc-pVTZ:B3LYP/3-21G. Only the central molecule was optimized, whereas the surrounding 16 asymmetric units were kept at fixed positions. Normal modes were calculated and transformed to Cartesian atomic displacements after optimization.

On the basis of the discussion by Dunitz *et al.* (1988 ▶), the temperature dependence of atomic motion can be described in analogy to a Boltzmann-type distribution of the harmonic oscillator. Atomic motion at higher temperatures can therefore be estimated by the formula given by Blessing (1995 ▶):
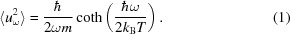
Although the molecular Einstein approach underlying the MO/MO calculations is not able to take into account lattice vibrations with acceptable accuracy, such a cluster calculation can provide a H/parent-atom 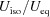 ratio, which is however dominated by internal atomic motion. Estimates so derived predict a higher ratio than the experimentally observed ratios from neutron and X-ray diffraction and require a 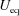 scale factor. To reach agreement between theory and experiment, and to take the temperature dependence into account, it was therefore necessary to go back to the TLS+ONIOM approach (Whitten & Spackman, 2006 ▶) and to include the experimental TLS contribution, treating the whole asymmetric unit as a rigid body. In this process the internal atomic relative displacement predicted by the MO/MO computation was subtracted from the experimental ADP data at a given temperature prior to the TLS fit (Schomaker & Trueblood, 1968 ▶). Both TLS fit and subtraction were performed by the program *APD-TOOLKIT*. A more sophisticated (and computationally more demanding) theoretical method based on periodic computations of different-sized unit-cell assemblies was studied by Madsen *et al.* (2013 ▶); for reproducing temperature dependence the TLS+ONIOM approach was sufficient.

## 4. Results and discussion   

### 4.1. Temperature dependence of *U*_iso_/*U*_eq_ of riding hydrogen and parent atom from X-ray data   

Prior to further analysis, a way to distinguish H atoms and their chemical environment is required. One choice would be the well established *SHELXL* AFIX groups. However, this would not distinguish H atoms exhibiting a distinct vibrational behaviour in theoretical calculations, *e.g.* an OH group in ethyl alcohol and one in phenol. The invariom formalism (Dittrich *et al.*, 2013 ▶) allows a finer distinction. Here H atoms that share the same invariom name are in the same covalent bonding environment and have the same number of next-nearest non-H neighbours, so it was used for classification throughout.^7^ Vibrational modes of individual invarioms (as derived from their model compounds) in other molecules will be investigated in a forthcoming study.

We can now consider the ratio of hydrogen 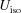 and parent-atom 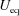 from X-ray diffraction at different temperatures. Initial observations with invariom refinements on d,l-serine (Dittrich *et al.*, 2005 ▶) indicated a temperature dependence at very low temperatures. Subsequent tests using the IAM showed that the IAM does not provide the model precision required to obtain significant results (Thorn, 2012 ▶). Our first question was therefore whether the 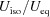 ratios from aspherical-atom refinements on NAC

**Figure 3:**
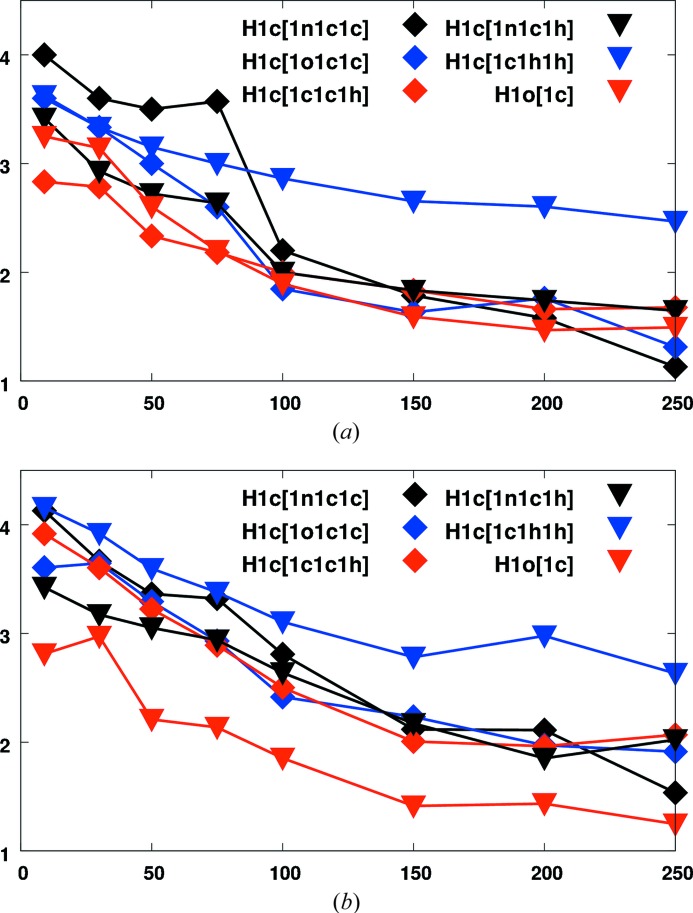
Temperature dependence of the 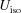/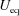 ratio from the X-ray data of *N*-acetyl-l-hydroxyproline monohydrate. (*a*) Invariom refinement with constrained hydrogen positions and refined 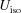. (*b*) Hirshfeld-atom refinement with freely refined hydrogen positions/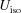, both using the same X-ray diffraction data.

H_2_O are able to reproduce the temperature dependence seen for d,l-serine. Fig. 3 ▶ shows such ratios for several hydrogen invarioms. Values were either obtained from invariom refinements with constrained riding hydrogen positions, but adjusted hydrogen 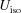 (Fig. 3 ▶ *a*), or by free least-squares refinement of positional and isotropic displacement parameters with HAR (Jayatilaka & Dittrich, 2008 ▶) (Fig. 3 ▶ *b*). As one can see, the programs/methods used, *XD* (Fig. 3 ▶ *a*) (Volkov *et al.*, 2006 ▶) and *TONTO* (Jayatilaka & Grimwood, 2003 ▶) (Fig. 3 ▶ *b*), give comparable results. Both refinements do indeed show the expected temperature dependence and even distinguish different hydrogen invarioms from X-ray data, although the standard deviation associated with each value is non-negligible (not shown for clarity).^8^ At very low temperatures the relative motion of H atoms relative to their parent atoms is clearly appreciably extended compared to higher temperatures.

This temperature dependence can be understood by looking at the low- and high-temperature limits of equation (1) as well as the transition temperature between both limits. Such dependence can be understood by considering the evolution of ADPs of atoms of different mass with temperature (Bürgi & Capelli, 2000 ▶). Division of the mass- and temperature-dependent functions 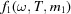 and 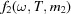 with masses 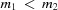 yields a function with the observed shape. Vibrations of atoms with larger contributions from higher internal frequencies are more prominent at lower temperatures, while at higher temperatures the atomic mass independent external modes dominate the overall amplitudes.

Interestingly, the data set that was an outlier in the expansion of the unit-cell volume at 67–75 K shows further deviations in atomic displacements: hydrogen invarioms of the type H1c[1c1h1h] mainly show a deviating behaviour with respect to the other atoms at higher temperatures. This is due to rotational disorder of the methyl group, and it is easily conceivable that the differences in the lattice constants seen at 67–75 K are due to the rotation becoming more frequent, either starting from this temperature or due to temperature fluctuations at this data point. We have previously studied similar disorder in methylaminobutyric acid hydrochloride by difference electron-density plots and molecular-dynamics simulations (Dittrich *et al.*, 2009 ▶). The abnormal temperature dependence of the three H1c[1c1h1h] methyl-hydrogen invarioms is proof that rotational disorder is also present in the acetyl group of NAC

H_2_O. We will study rotational disorder in this molecule and its anhydrous form in more detail in a subsequent study.

Since positional and displacement parameters are correlated, limiting model flexibility (constrained hydrogen positions) seems to make the onset of additional rotational motion more apparent in invariom refinement, whereas free refinement of positions and 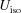 seems to lead to an over-parameterized model in HAR.

### 4.2. QM/MM and MO/MO calculations   

We were interested in reproducing the temperature dependence of 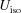 by using the TLS+ONIOM approach (Whitten & Spackman, 2006 ▶). For this purpose the above-mentioned two-layer ONIOM computation using the coordinates from the 9 K X-ray diffraction experiment was combined with a TLS fit at each temperature to provide another set of results that includes information independent from experiment.

Details of the procedure need to be highlighted prior to a discussion of the results. Before performing the TLS fit the computed internal modes were subtracted from the experimental non-H-atom ADPs. Contrary to expectation, this is not accompanied by an improvement of the TLS *R* factors (not shown) with temperature, since the internal ADPs of heavy atoms are mostly spherical in shape and get almost completely absorbed in the TLS ADPs. Nevertheless, such a correction is physically reasonable and we recommend that it is performed. Furthermore, the agreement of the ratio of 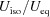 seen in the aspherical-atom refinements of the X-ray data improves when this internal TLS contribution is taken into account.

Another detail concerns the low-frequency modes describing the movement of the asymmetric unit in the crystal framework. Low-frequency modes have a very large impact on the overall displacements, which can be derived directly from equation (1). Since the approximations present in the theoretical method do not allow the estimation of these frequencies with sufficient accuracy, low-frequency modes were omitted in the calculation of ADPs. A frequency threshold of 200 cm^−1^ was found to be adequate (Madsen *et al.*, 2013 ▶). The required information on the overall displacement is instead taken from the TLS fit, which yields more reliable values. The TLS+ONIOM approach is hence an attractive computational method to understand the ratio of H-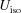/parent 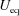 when experimental TLS contributions are available. It reproduces the temperature dependence nicely, although rotational disorder cannot be predicted. More work is required for *ab initio* prediction of the temperature dependence by theoretical computations without any experimental input. So far it can only provide an independent source of information for the internal modes and requires the application of the TLS fit; theoretical methods are nevertheless best suited to provide H-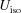/*X*-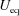 ratios since experimental errors are limited to ADPs used in the TLS fit.

**Figure 4:**
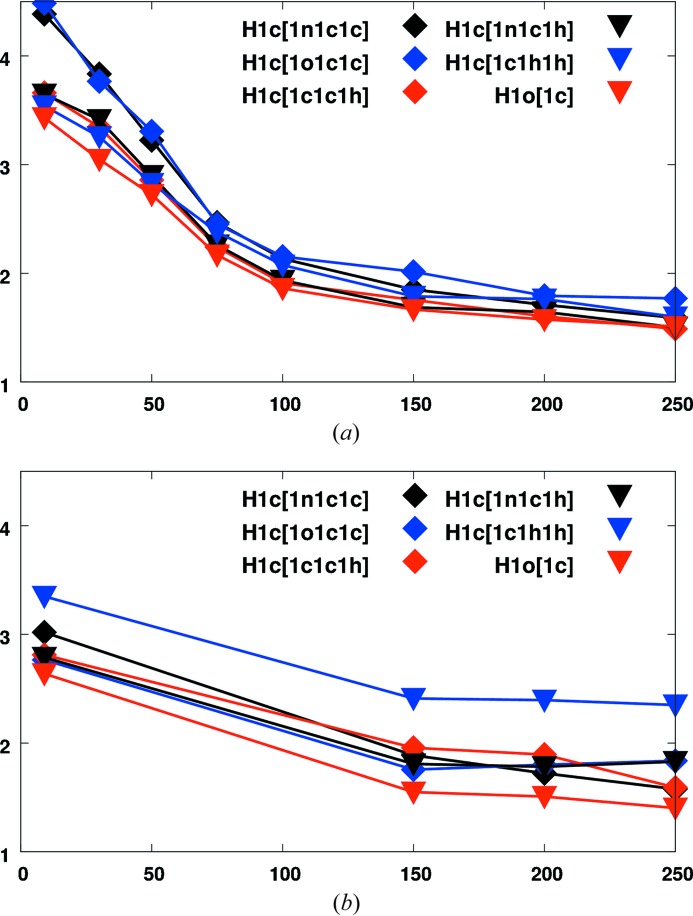
Temperature dependence of the 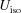/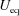 ratios obtained by TLS+ONIOM and neutron diffraction. (*a*) Internal vibrations from MO/MO ONIOM calculations and TLS fit against ADPs obtained by invariom refinement. (*b*) Refinement against neutron diffraction data.

We now compare these TLS+ONIOM results to those from neutron diffraction, our experimental benchmark (Fig. 4 ▶). Since neutron diffraction data sets were not collected at temperatures of 30, 50, 75 and 100 K, these data points are absent in the comparison with high-resolution X-ray and TLS+ONIOM results. The TLS+ONIOM approach confirms that individual displacements of hydrogen invarioms are distinguishable mainly at temperatures below 100 K. Rotational disorder cannot be predicted from this approach, whereas it is also detectable in the neutron data at higher temperatures. Good agreement of neutron diffraction and the TLS+ONIOM approach is found for the temperature-dependent ratio of hydrogen 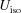 or 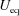 and the parent atom, which also agrees rather well with the X-ray results in Fig. 3 ▶. However, at higher temperatures HAR and neutron diffraction show a trend that deviates from the other methods, with the ratio being higher than 1.5, whereas the ratio is smaller in invariom refinement. This is probably due to the predicted/constrained hydrogen positions in invariom refinement, which impose a beneficial limit on the flexibility of the structural model here. Since the rigid-body fit in the TLS+ONIOM approach is based on the invariom results, a similar temperature dependence to that in invariom refinement is observed.

A comparison of Figs. 3 ▶ and 4 ▶ indicates an overall surprisingly good agreement for each curve of H-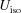/*X*-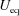 *versus* temperature over the whole range, especially when taking into account that different methods/experiments were used. All four methods consistently indicate that at very low temperatures the ratio H-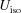/*X*-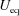 can be as high as four, *e.g.* for H atoms attached to an *sp* ^3^ C atom with three non-H-atom neighbours (corresponding to AFIX 13 in *SHELXL*). Moreover, all four methods consistently confirm (or reproduce in the case of TLS+ONIOM) the temperature dependence that is predicted from equation (1). Conventional IAM structure determinations employing riding hydrogen constraints – and likewise models with aspherical scattering factors – should therefore take the temperature-dependent ratio into account.

## 5. Conclusion and outlook   

Four different methods providing the temperature-dependent ratio of H-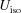 to *X*-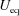 in the riding hydrogen treatment have been evaluated and compared. Neutron diffraction experiments provide benchmark values. ‘Invariom’ and ‘Hirshfeld-atom’ aspherical-atom refinements with high-resolution X-ray diffraction data yield very similar results, with the invariom model using constrained hydrogen positions giving a more consistent result, but the Hirshfeld-atom model being closer to neutron diffraction at higher temperatures. Implementing restraints in the *TONTO* program would therefore be useful. Furthermore, experimental findings can be well reproduced by the TLS+ONIOM approach. Here a single quantum chemical MO/MO cluster calculation is combined with a temperature-dependent rigid-body fit of the non-hydrogen ADPs from aspherical-atom X-ray refinements. All methods show that the ratio of H-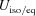/*X*-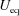, which is usually assumed to be 1.2 or 1.5 independent of temperature, is frequently more than twice as high at lower temperatures. Fixed values of 1.2 or 1.5, as usually used in conventional spherical-atom ‘IAM’ refinements, are therefore underestimating the relative displacement of H atoms at cryogenic temperatures. Since all methods used here consistently show or reproduce that the H-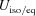/*X*-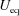 ratio is temperature dependent, the effect should be taken into account in low-temperature structure determinations, especially around 100 K and below. We will provide relevant functionality (program *APD-TOOLKIT*) in subsequent work.

## Supplementary Material

Crystal structure: contains datablock(s) 9K, 30K, 50K, 75K, 100K, 150K, xcalibur, 250K, hydroxyproline9K, hydroxyproline150K, hydroxyproline200K, hydroxyproline250K. DOI: 10.1107/S2053273314010626/kx5033sup1.cif

CCDC references: 990102, 990103, 990104, 990105, 990106, 990107, 990108, 990109, 990114, 990115, 990116, 990117, 977814, 977815, 977816, 977817

## Figures and Tables

**Table 1:** Crystal data of *N*-acetyl-L-4-hydroxyproline monohydrate from invariom refinements GoF = goodness of fit; GoFW = goodness of fit (weighted).

Crystal data								
Chemical formula	C_7_H_10_NO 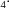 H_2_O							
Formula weight	191.18							
Cell setting, space group	Orthorhombic, 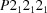							
Temperature (K)	9	30	50	75	100	150	200	250
*a* (Å)	9.854 (3)	9.853 (4)	9.866 (7)	9.884 (6)	9.9026 (2)	9.9408 (2)	9.9748 (2)	10.0123 (2)
*b* (Å)	9.249 (3)	9.251 (5)	9.250 (7)	9.253 (6)	9.2485 (2)	9.2479 (2)	9.2492 (2)	9.2556 (2)
*c* (Å)	10.144 (2)	10.145 (2)	10.149 (6)	10.155 (3)	10.1662 (2)	10.1875 (2)	10.2103 (2)	10.2441 (2)
*V* (Å^3^)	924.5 (4)	924.7 (7)	926.2 (11)	928.7 (9)	931.06 (3)	936.55 (3)	941.99 (3)	949.32 (3)
*Z*, *F*(000)	4, 408							
*D*_*x*_ (Mg m^−3^)	1.374	1.373	1.371	1.367	1.364	1.356	1.348	1.338
Radiation type	Synchrotron	Synchrotron	Synchrotron	Synchrotron	Mo *K*α	Mo *K*α	Mo *K*α	Mo *K*α
μ (mm^−1^)	0.070	0.061	0.061	0.061	0.116	0.116	0.115	0.114
Crystal form, colour	Rectangular, colourless				Rectangular, colourless			
Crystal size (mm)	0.34 × 0.28× 0.28				0.54 × 0.27 × 0.14			
								
Data collection								
Diffractometer	Huber Type 512				Oxford Diffraction Xcalibur S			
Data-collection method	ϕ scans				ω and ϕ scans			
Absorption correction	None				Analytical			
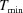 , 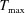	n/a	n/a	n/a	n/a	0.959/0.986	0.954/0.987	0.960/0.989	0.956/0.989
No. of measured reflections	45747	25178	44258	45127	39746	30837	30309	17876
No. of independent reflections	8304	7775	7803	7809	10866	7826	7829	4420
No. of observed reflections	7885	7262	7372	7255	7744	5673	4860	3245
Criterion for observed reflections	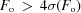							
*R*_int_ (%)	0.040	0.038	0.055	0.051	0.037	0.039	0.039	0.020
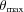 (°), 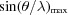	31.90, 1.022	31.88, 1.000	31.90, 1.000	31.87, 1.000	53.28, 1.132	53.32, 1.000	53.31, 1.069	36.25, 0.833
								
Invariom refinement								
Refinement on	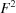							
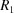 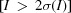	0.026	0.028	0.026	0.028	0.031	0.029	0.029	0.025
No. of reflections	7885	7262	7372	7255	7744	5673	4860	3245
No. of parameters	131							
H-atom treatment	Invarioms: calculated H position, bond-length elongated, 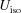 refined; HAR: all parameters adjusted							
Weighting scheme								
1/ 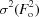 + [] (*P* = 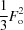 + 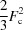 )	[0.06 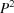 + 0.04*P*]	[0.04 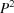 + 0.05*P*]	[0.04 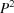 + 0.04*P*]	[0.05 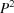 + 0.02*P*]	[0.04 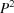 + 0.07*P*]	[0.05 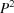 + 0.05*P*]	[0.06 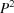 + 0.04*P*]	[0.04 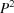 + 0.08*P*]
GoF	1.76	1.44	1.48	2.02	2.81	2.96	2.12	3.84
GoFW	0.96	0.95	0.94	1.00	0.81	0.84	0.82	0.81
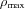 , 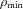 (e Å^−3^)	0.36/−0.25	0.32/−0.22	0.27/−0.21	0.30/−0.25	0.36/−0.21	0.25/−0.16	0.20/−0.17	0.16/−0.12

**Table 2:** Scattering factor assigned during invariom refinement with atom names, invariom names, local atomic site symmetry and model compounds they were derived from

Atom name	Invariom name	Local atomic site symmetry	Model compound
O1	O2c	*mm*2	Formaldehyde
O2	O1c1h	*m*_*z*_	Methanol
O3	O1.5c[1.5n1c]	*mm*2	Acetamide
O4	O1c1h	*m*_*z*_	Methanol
O5	O1h1h	*mm*2	Water
N1	N1.5c[1.5o1c]1c1c	*m*_*z*_	*N*,*N*-Dimethylacetamide
C1	C2o1o1c	*m*_*z*_	Acetic acid
C2	C1n1c1c1h	*m*_*z*_	2-Aminopropane
C3	C1c1c1h1h	*mm*2	Propane
C4	C1o1c1c1h	*m*_*z*_	2-Propanol
C5	C1n1c1h1h	*m*_*z*_	Ethylamine
C6	C1.5o1.5n[1c1c]1c	*m*_*z*_	*N*,*N*-Dimethylacetamide
C7	C1c1h1h1h	3*m*	Ethane
H1,2	H1o[1c]	6	Methanol
H3	H1c[1n1c1c]	6	2-Aminopropane
H4,5	H1c[1c1c1h]	6	Propane
H6	H1c[1o1c1c]	6	2-Propanol
H7,8	H1c[1n1c1h]	6	Ethylamine
H9,10,11	H1c[1c1h1h]	6	Ethane
H12,13	H1o[1h]	6	Water

## Footnotes

1 This was only tested for scattering factors from the generalized invariom database (Dittrich *et al.*, 2013 ▶), not for those from the UBDB2011  (Jarzembska & Dominiak, 2012 ▶), the ELMAM2  (Domagala *et al.*, 2012 ▶) nor the SBFA (Hathwar *et al.*, 2011 ▶) libraries. For modelling hydrogen scattering, theoretically derived databases have the advantage of higher precision, since experimental scattering factors for hydrogen can only be reliably determined to the dipolar level of the multipole expansion.

2 Since displacement parameters used in this work are either isotropic or anisotropic, we use the abbreviation ADPs, which was recommended to be used only for anisotropic displacement parameters (Trueblood *et al.*, 1996 ▶), in a different manner here.

3 Post-analysis of the temperature and volume dependence of unit-cell parameters showed that the data point at 67 K (as indicated on the low-*T* device) was an outlier, probably due to inaccuracies caused by heating the cold stream of He gas to higher temperatures. We have corrected this temperature to 75 K, as derived from a plot of the increase of the unit-cell volume with temperature. Another reason for the deviating behaviour might be rotational disorder and this is discussed later on.

4 This new segmented-body TLS refinement program and its functionality will be described in a forthcoming paper.

5 We have also refined isotropic ADPs for H atoms using the neutron data instead of the anisotropic description. Results show the same trends within the standard deviations of our experiments, but since the figures of merit are worse we chose to use H- for neutron data and H-for the X-ray data.

6 Supporting information is available from the IUCr electronic archives (Reference: KX5033).

7 It should be noted that differences due to hydrogen bonding are not taken into account in the invariom approach. However, such influences are an order of magnitude smaller than electron-density redistributions due to covalent bonding, which are not taken into account in the IAM model.

8 For the most common invariom H1c[1c1h1h], which is used three times, the standard deviation of H/*X*  varies from 0.03 to 0.3 across all temperatures and experiments.

## References

[bb1] Betteridge, P. W., Carruthers, J. R., Cooper, R. I., Prout, K. & Watkin, D. J. (2003). *J. Appl. Cryst.* **36**, 1487.

[bb2] Blessing, R. H. (1995). *Acta Cryst.* B**51**, 816–823. 10.1107/s0108768194012474 7576375

[bb3] Bürgi, H. B. & Capelli, S. C. (2000). *Acta Cryst.* A**56**, 403–412. 10.1107/s0108767300005626 10967519

[bb4] Burnett, M. N. & Johnson, C. K. (1996). *ORTEPIII* Report ORNL-6895. Oak Ridge National Laboratory, Oak Ridge, Tennessee, USA.

[bb5] Busing, W. R. & Levy, H. A. (1964). *Acta Cryst.* **17**, 142–146.

[bb102] Capelli, S. C., Bürgi, H.-B., Dittrich, B., Grabowsky, S. & Jayatilaka, D. (2014). *IUCrJ* Submitted. 10.1107/S2052252514014845 PMC4174878 25295177

[bb6] Checińska, L., Morgenroth, W., Paulmann, C., Jayatilaka, D. & Dittrich, B. (2013). *CrystEngComm*, **15**, 2084–2090.

[bb7] Clark, R. C. & Reid, J. S. (1995). *Acta Cryst.* A**51**, 887–897.

[bb8] Dittrich, B., Hübschle, C. B., Luger, P. & Spackman, M. A. (2006). *Acta Cryst.* D**62**, 1325–1335. 10.1107/S090744490602899X 17057335

[bb9] Dittrich, B., Hübschle, C. B., Messerschmidt, M., Kalinowski, R., Girnt, D. & Luger, P. (2005). *Acta Cryst.* A**61**, 314–320. 10.1107/S0108767305005039 15846034

[bb10] Dittrich, B., Hübschle, C. B., Pröpper, K., Dietrich, F., Stolper, T. & Holstein, J. J. (2013). *Acta Cryst.* B**69**, 91–104. 10.1107/S2052519213002285 23719696

[bb11] Dittrich, B., Koritsánszky, T. & Luger, P. (2004). *Angew. Chem. Int. Ed.* **43**, 2718–2721. 10.1002/anie.200353596 18629999

[bb12] Dittrich, B., McKinnon, J. J. & Warren, J. E. (2008). *Acta Cryst.* B**64**, 750–759. 10.1107/S0108768108032163 19029704

[bb13] Dittrich, B., Pfitzenreuter, S. & Hübschle, C. B. (2012). *Acta Cryst.* A**68**, 110–116. 10.1107/S0108767311037974 22186287

[bb14] Dittrich, B., Warren, J. E., Fabbiani, F. P., Morgenroth, W. & Corry, B. (2009). *Phys. Chem. Chem. Phys.* **11**, 2601–2609. 10.1039/b819157c 19421516

[bb15] Domagała, S., Fournier, B., Liebschner, D., Guillot, B. & Jelsch, C. (2012). *Acta Cryst.* A**68**, 337–351. 10.1107/S0108767312008197 22514066

[bb16] Dunitz, J. D., Schomaker, V. & Trueblood, K. N. (1988). *J. Phys. Chem.* **92**, 856–867.

[bb17] Dunning, T. H. (1989). *J. Chem. Phys.* **90**, 1007–1023.

[bb101] Edwards, A. J. (2011). *Aust. J. Chem.* **64**, 869–872.

[bb18] Fischer, R. X. & Tillmanns, E. (1988). *Acta Cryst.* C**44**, 775–776.

[bb19] Hansen, N. K. & Coppens, P. (1978). *Acta Cryst.* A**34**, 909–921.

[bb20] Hathwar, V. R., Thakur, T. S., Row, T. N. G. & Desiraju, G. R. (2011). *Cryst. Growth Des.* **11**, 616–623.

[bb21] Hirshfeld, F. L. (1977). *Theor. Chim. Acta (Berl.)*, **44**, 129–138.

[bb22] Hospital, M., Courseille, C. & Leroy, F. (1979). *Biopolymers*, **18**, 1141–1148.

[bb23] Hübschle, C. B., Luger, P. & Dittrich, B. (2007). *J. Appl. Cryst.* **40**, 623–627.

[bb24] Jarzembska, K. N. & Dominiak, P. M. (2012). *Acta Cryst.* A**68**, 139–147. 10.1107/S0108767311042176 22186290

[bb25] Jayatilaka, D. (1994). *Chem. Phys. Lett.* **230**, 228–230.

[bb26] Jayatilaka, D. & Dittrich, B. (2008). *Acta Cryst.* A**64**, 383–393. 10.1107/S0108767308005709 18421128

[bb27] Jayatilaka, D. & Grimwood, D. J. (2003). *Computational Science – ICCS 2003*, edited by P. M. A. Sloot, D. Abramson, A. V. Bogdanov, J. J. Dongarra, A. Y. Zomaya & Y. E. Gorbachev. *Lecture Notes in Computer Science*, Vol. 2660, pp. 142–151. Heidelberg: Springer.

[bb28] Jelsch, C., Pichon-Pesme, V., Lecomte, C. & Aubry, A. (1998). *Acta Cryst.* D**54**, 1306–1318. 10.1107/s0907444998004466 10089507

[bb29] Johnas, S. K. J., Morgenroth, W. & Weckert, E. (2006). *Jahresber. HASYLAB*, pp. 325–328. Hamburg: DESY.

[bb30] Kabsch, W. (2010). *Acta Cryst.* D**66**, 125–132. 10.1107/S0907444909047337 PMC2815665 20124692

[bb31] Madsen, A. Ø. (2006). *J. Appl. Cryst.* **39**, 757–758.

[bb32] Madsen, A. Ø., Civalleri, B., Ferrabone, M., Pascale, F. & Erba, A. (2013). *Acta Cryst.* A**69**, 309–321.

[bb33] Munshi, P., Madsen, A. Ø., Spackman, M. A., Larsen, S. & Destro, R. (2008). *Acta Cryst.* A**64**, 465–475. 10.1107/S010876730801341X 18560163

[bb34] Oxford Diffraction Ltd (2006). *CrysAlis CCD and CrysAlis RED* Oxford Diffraction Ltd, Oxford, England.

[bb35] Piltz, R. (2011). *Acta Cryst.* A**67**, C155.

[bb36] Pröpper, K., Holstein, J. J., Hübschle, C. B., Bond, C. S. & Dittrich, B. (2013). *Acta Cryst.* D**69**, 1530–1539. 10.1107/S0907444913010664 23897476

[bb37] Schomaker, V. & Trueblood, K. N. (1968). *Acta Cryst.* B**24**, 63–76.

[bb38] Schürmann, C. J., Pröpper, K., Wagner, T. & Dittrich, B. (2012). *Acta Cryst.* B**68**, 313–317. 10.1107/S0108768112017831 22610682

[bb39] Sheldrick, G. M. (2008). *Acta Cryst.* A**64**, 112–122. 10.1107/S0108767307043930 18156677

[bb40] Stewart, R. F. (1976). *Acta Cryst.* A**32**, 182–185.

[bb41] Thorn, A. (2012). Personal communication.

[bb100] Trueblood, K. N., Bürgi, H.-B., Burzlaff, H., Dunitz, J. D., Gramaccioli, C. M., Schulz, H. H., Shmueli, U. & Abrahams, S. C. (1996). *Acta Cryst.* A**52**, 770–781.

[bb42] Volkov, A., Macchi, P., Farrugia, L. J., Gatti, C., Mallinson, P., Richter, T. & Koritsánszky, T. (2006). *XD2006 – a Computer Program Package for Multipole Refinement, Topological Analysis of Charge Densities and Evaluation of Intermolecular Energies from Experimental or Theoretical Structure Factors.*

[bb43] Whitten, A. E. & Spackman, M. A. (2006). *Acta Cryst.* B**62**, 875–888. 10.1107/S0108768106020787 16983168

[bb103] Woinska, M., Jayatilaka, D., Spackman, M. A., Edwards, A. J., Dominiak, P. M., Wozniak, K., Nishibori, E., Sugimoto, K. & Grabowsky, S. (2014). *Acta Cryst.* A. Submitted. 10.1107/S2053273314012443 25176996

[bb44] Wu, G., Rodrigues, B. L. & Coppens, P. (2002). *J. Appl. Cryst.* **35**, 356–359.

